# Debilitating Darier’s Disease and Its Impact on the Quality of Life

**DOI:** 10.7759/cureus.8133

**Published:** 2020-05-15

**Authors:** Prashanth Ashok Kumar, Shweta Paulraj, Sakshi Dutta

**Affiliations:** 1 Internal Medicine, State University of New York Upstate Medical University, Syracuse, USA

**Keywords:** darier disease, genodermatosis, heath related quality of life, retinoids

## Abstract

Darier's disease (DD) is a rare, autosomal dominant genodermatosis that occurs due to mutations in the ATP2A2 gene on chromosome 12q23-24 that codes for sarco/endoplasmic reticulum calcium ATPase (SERCA), causing desmosomal breakdown and acantholysis. The disease usually persists for life and is characterized by a relapsing-remitting course. It can be challenging to treat and can cause several complications that may result in frequent hospitalizations. Sepsis, with the damaged skin as the portal of entry, and widespread herpes can occur. Studies have shown genetic links and associations between DD and psychiatric conditions like recurrent depression and bipolar disorder. We report the case of a 70-year-old male with a severe form of the condition and highlight his clinical course. We describe the severe nature of his disease that resulted in multiple complications like recurrent bacterial skin infections, significantly impairing his quality of life and leading to his ultimate demise.

## Introduction

Darier's disease (DD) is also known as keratosis follicularis, dyskeratosis follicularis, and Darier White disease. The disease was first described in 1886 by Price Marrow and then reported in 1889 by both Darier and White independently. It is an inherited genodermatosis with implications ranging from cosmetic to severe functional impairment, depending on the severity [1,2]. We describe the case of an elderly gentleman with DD who experienced frequent hospital admissions and debilitating complications resulting in functional impairment and eventual demise.

## Case presentation

Our patient was a 70-year-old Caucasian male with an established diagnosis of DD since his childhood years. He had had innumerable exacerbations in the past and multiple admissions for secondary bacterial infections. He presented to the hospital with worsening of coalescing, firm, greasy, and bright red to skin-colored macules and papules located around his gluteal clefts, back, scalp, and forehead causing severe debilitating pain. The gluteal lesions were the most clinically relevant symptom and they were causing profound distress. He had severe physical deconditioning and had fallen several times prior to his arrival. A few weeks before his hospitalization, he had been diagnosed with intraductal papillary mucinous neoplasm (IPMN) of the pancreas and had undergone a total pancreatectomy and splenectomy with hepaticojejunostomy and Billroth II gastrojejunostomy, which had contributed to his fatigue and weakness. He had no abdominal symptoms and his abdominal exam was initially benign. His vitals were stable, and there was no systemic or laboratory evidence of an infectious process at the time of admission. As the clinical course progressed, his skin lesions and clinical status continued to worsen. He developed bacteremia secondary to *Streptococcus dysgalactiae* and then* Pseudomonas* a few days later. A week later, he developed bacterial peritonitis with *Enterococcus faecalis, Klebsiella pneumoniae, Candida glabrata*, and *E. coli*. He received multiple courses of broad-spectrum antibiotics and antifungals. The dermatology service was closely following the patient and had started him on acitretin with a dose of up to 50 mg daily. He was also started on systemic steroids. Although he had intermittent days of temporary improvement and relief, his overall progress was mired with worsening skin lesions, sepsis causing hypotension, and functional decline.

Pain management, palliative care, infectious diseases, burn surgery, surgical ICU, social work, cardiology, and endocrinology were involved in his care and followed him closely. The patient had had recurrent exacerbations of his skin disease in the past, especially following a stressful event like surgery. Acitretin controlled it for a limited time, only to be followed by another exacerbation. He had suffered multiple exacerbations with superimposed S*treptococcal* and *Pseudomonas* cellulitis over the years. The patient felt physically and emotionally exhausted, and his quality of life was significantly impaired. He was also emotionally drained. He decided to adopt comfort measures alone and ultimately passed away within a few months.

## Discussion

DD is a rare, autosomal dominant genodermatosis with a prevalence ranging from one to three per 100,000 people. The disease occurs equally in both sexes and has been noted in most ethnic groups. Worldwide distribution has been reported with mild variation in prevalence in different regions [1-4]. It occurs due to mutations in the ATP2A2 gene on chromosome 12q23-24 that codes for sarco/endoplasmic reticulum calcium ATPase (SERCA) isoform 2 resulting in desmosome breakdown and acantholysis [5,6].

Manifestations usually begin at childhood or adolescence. Characteristic lesions are hyperkeratotic, erythematous, pruritic plaques that may ulcerate, scale and turn gray, get crusted, or coalesce into larger lesions. They may become extremely painful and foul-smelling, especially if there is a superimposed infection. Palmar and plantar keratosis, nail changes like fissuring, streaking, and subungual keratosis may be present. Oral and other mucosal lesions may be present in up to 15-20% of the patients. Distinct clinical variants have been described by some authors. These include hypertrophic, vesiculobullous, cornifying, comedonal, and zosteriform or linear lesions. The clinical course may be dominated by skin fragility and painful erosions as seen in our patient. Abnormal cell-to-cell adhesion and aberrant keratinization are the hallmark histological features. Some authors have suggested that histological features correlate with clinical manifestations and disease severity [1,7]. Other academics have classified the disease based on both molecular and clinical features such as the acral hemorrhagic and segmental subtypes. In the former, the ATP2A2 gene mutation occurs in the vascular endothelium in addition to the keratinocytes. Hemorrhagic vesicles are usually seen in the hands. In the latter, segmental lesions are noted along the lines of skin cell development and are characterized by somatic mosaicism in the ATP2A2 gene [7].

The disease usually persists for life and has no cure. Treatment is never complete, and the disease is characterized by a relapsing-remitting course [6]. Topical agents like retinoids, tazarotene, and 5-fluorouracil have been shown to benefit DD that is limited and not severe. More severe presentations, like in our patient, usually require high-dose retinoids or a prolonged course of low-dose retinoids. Steroids may provide symptomatic benefit. Avoiding triggers like sunlight, heat, occlusive clothing, and friction may prevent exacerbations. The disease can be partially controlled by the above measures but no permanent cure exists [1,5,6]. Recent evidence has suggested that the use of low-dose intravenous immunoglobulin (IVIG) may be beneficial in recurrent DD. Legrand et al. treated a 54-year-old male with 0.4 g/kg IVIG every three weeks and noted significant improvement after four courses of treatment. IVIG may improve the immune response and help prevent superimposed skin infections [5]. Leung et al. have reported three cases of severe recalcitrant DD treated with photon and electron beam radiation therapy, which resulted in long-term relief [8]. Although new treatment options are emerging, no large-scale studies are available to effectively guide physicians in the treatment [1].

The condition is challenging to control and treat and results in complications that may cause frequent hospitalizations. Recurrent skin infections are one such compilation. They may be self-limited, or life-threatening with sepsis. The infection superimposed on the already painful widespread skin lesions causes patients to suffer from severe pain, impairing their quality of life. Some reports suggest that patients with DD have high *Staphylococcus aureus* colonization in the skin and nares, which may correlate with the disease severity. However, more research is required to establish the same. Widespread herpes coinfection and endophthalmitis have also been reported [9,10].

Patients with DD are at an elevated risk of neuropsychiatric manifestations. A study in the UK involving 100 unrelated individuals with DD, mostly Caucasian, showed that there was a high lifetime incidence of mood disorders like major depression, bipolar disorder, and suicidal behavior among DD patients compared to the general population [11]. Schizophrenia has been associated with DD and has also been reported in non-Caucasian races [12]. Studies attempting to establish a genetic linkage to such manifestations have been reported but have remained controversial [13,14].

As seen in our patient and in other cases reported so far, patients with DD suffer from several physical and psychological distress and complications that result in morbidity and mortality. Through our case, we hope to raise awareness of this rare disease and the need for a holistic approach in managing the aspects needed in the care of these patients. More studies and guidance are needed so as to better manage this debilitating disorder.

An abstract of the case was accepted for presentation at the Society of Hospital Medicine Annual Conference 2020 (HM20). The abstract is scheduled to be published in the online supplement of the Journal of Hospital Medicine (Abstract: Ashok Kumar P, Paulraj S, Dutta S. Debilitating Darier’s Disease Causing Recurrent Bacterial Infections and Its Impact on Quality of Life. The Need for a Multidisciplinary Approach. Journal of Hospital Medicine; May 2020). A poster of the abstract is scheduled to be presented at the Virtual Poster Competition of HM20 (Poster: Ashok Kumar P, Paulraj S, Dutta S. Debilitating Darier’s Disease Causing Recurrent Bacterial Infections and Its Impact on Quality of Life. The Need for a Multidisciplinary Approach. SHM Annual Meeting 2020; July 2020).

## Conclusions

DD is a rare, inherited skin disorder causing frequent inpatient admissions for those affected by it. Hospitalists and care providers need to be aware that these patients require a multidisciplinary approach involving primary care providers, dermatologists, infectious disease specialists, pain management specialists, palliative care experts, psychiatrists, and social workers as the disease can have profound effects on both the physical and mental wellbeing of the patients.
